# Contribution of Slovenian community pharmacist counseling to patients’ knowledge about their prescription medicines: a cross-sectional study

**DOI:** 10.3325/cmj.2015.56.41

**Published:** 2015-02

**Authors:** Nejc Horvat, Mitja Kos

**Affiliations:** Chair of Social Pharmacy, University of Ljubljana – Faculty of Pharmacy, Ljubljana, Slovenia

## Abstract

**Aim:**

To assess patients’ knowledge about prescription medicines they are taking and their view on how much community pharmacist counseling contributed to their knowledge.

**Methods:**

An observational study was designed to obtain information about patients’ knowledge, their view on pharmacist counseling, and physicians’/pharmacists’ provision of information. This study used a specifically designed questionnaire, which served as an interview guide. 400 patients picking up a prescription medicine were structurally interviewed upon leaving one of the 20 randomly chosen Slovenian pharmacies. The interviews took place in November and December 2013.

**Results:**

Patients were familiar with general information about the medicines and their application (93%-100% of patients). Knowledge about considerations (16% of patients) and adverse effects (20% of patients) was limited. Factors associated with patient knowledge were physicians’/pharmacists’ adequate provision of information (β = 0.259), patient’s age (β = - 0.149), patient’s education (β = 0.100), and prescription type (β = -0.104). Patients’ responses were mostly consistent with the Summaries of Product Characteristics (72%-96% of responses). However, 42% of responses to the question about taking medicine with meals were incorrect. Pharmacists routinely informed the patients about medication purpose, dose, application rate, and timing of medication (in 72%, 89%, 89%, and 77% of cases, respectively). Other information was rarely offered. Patients with new prescriptions received significantly more counseling (pharmacist counseling score 5.9, 5.2, and 4.7 of maximum 10 for new, regular, and refill prescriptions, respectively, *P* = 0.001) and obtained adequate labeling (69%, 26%, and 17% of patients for new, regular and refill prescriptions, respectively, *P* < 0.001) than patients with regular or refill prescriptions.

**Conclusion:**

Patients were familiar with basic information about administration of their prescription medicines, but lacked knowledge about medication safety. This could be attributed to pharmacist counseling, which primarily focused on medicine use instructions.

The number of prescriptions dispensed in Slovenian community pharmacies has gradually increased, with more than 16.5 million prescriptions in 2013 and an average of 8 prescriptions per inhabitant per year (1). Patients, particularly those who use multiple medicines, are likely to experience drug-related problems, which might increase morbidity, mortality, and hospital admissions, all having substantial economic impact (2-5).

Patient education ensures optimal use of medicines and minimizes drug-related problems. Patients’ knowledge enhances active participation in therapy, thus increasing adherence and ultimately leading to better treatment outcomes (6-10). A fundamental source of patient education about medicines are community pharmacists, as they typically offer the last health professional advice before patients start taking their medicines. Therefore, their responsibility is to provide medication counseling every time they dispense a prescription medicine. Effective counseling includes two fundamental processes: asking patients what they already know and filling in knowledge gaps (11,12).

Community pharmacy services in Slovenia are provided by either public pharmaceutical institutions (founded by local authorities) or privately-owned pharmacies. Patients can collect their prescription medicines in any of the pharmacies. At the end of 2013, there were 319 community pharmacies (pharmacy density of 6455 inhabitants per pharmacy) and 52 active pharmacists per 100 000 inhabitants (13,14). Costs of prescription medicines are covered by a combination of the compulsory and additional voluntary health insurance (15). All pharmacies are members of the Chamber, which serves as a supervisory body.

Good pharmacy counseling improves patients’ knowledge and their use of medicines (7,8,10). Several observational studies evaluated quality of counseling by focusing on the counseling process or patients’ knowledge (6,11,16-21). However, none of these studies attempted to relate patient knowledge and pharmacist counseling as viewed by patients. The aim of the current study was to assess patients’ knowledge about prescription medicines they are taking and to identify their view of how much community pharmacist counseling contributed to their knowledge.

## Participants and methods

This cross-sectional survey used structured interviews with patients exiting randomly chosen pharmacies. Ethical approval for the study was obtained from the National Medical Ethics Committee.

### Questionnaire design

This study used a specifically designed questionnaire (Supplementary material)(web extra material 1), which served as an interview guide. The content of the questionnaire was derived from pharmacy counseling literature (22-24). The questionnaire comprised 10 open-ended questions dealing with patients’ knowledge about medications. The first 8 questions focused on specific counseling elements regarding the medication: purpose, dose, application rate, timing, route of administration, taking with meals, duration of therapy, and recognition of medication effectiveness. The last 2 were general questions about considerations and adverse effects (ie, “What do you know about …?”). Therefore, responses to the question about considerations could include interactions, contraindications, precautions, warnings, and so on. Responses to the question about adverse effects included adverse effects, associated time frames, how to prevent or minimize adverse effects, and so on.

The ten open ended questions were followed by 3 yes/no questions. These examined patients’ view on pharmacists’ contribution to their knowledge about a specific counseling element. The first question asked whether the pharmacist informed them about a specific counseling element at their most recent pharmacy visit. If the answer was negative, the second question asked whether the pharmacist specifically checked if the patient already possessed this knowledge. The third question addressed the patients’ cumulative experiences with their physicians and pharmacists, asking if their health care providers adequately informed them about a specific counseling element. In this respect, information needs were identified. The question included physicians in case patients did not remember who exactly provided that type of information during their treatment. The last question addressed any other, so far unexpressed, important medication information that the pharmacists provided during the most recent pharmacy visit. The provision of a medication label or any other written information was also investigated.

Other medication-specific data included the name, dosage strength, and prescription type (new, regular, refill). New prescriptions were those written for first-time users. Regular prescriptions were prescriptions for the medicines that the patient previously used, but with no refills. Refill prescriptions were prescriptions for the medicines that the patient previously used, with refills. Other patient-specific data included age, sex, education, income, patient’s current number of prescription medicines, patient’s assessment of current health status, and patient’s assessment of pharmacist counseling. Relevant experts with experience in pharmacy practice were consulted regarding the content of the questionnaire. Afterwards, the drafted questionnaire was tested in a pilot study on a convenient sample of patients to establish its face and content validity.

### Sample

The exit survey involved patients leaving a community pharmacy. The sample had to be large enough to provide 95% confidence interval of a single proportion with a span of 0.1 (from -0.05 to +0.05), assuming a proportion of 0.5 (50% counseling rate). The calculated required sample size was 385, which was rounded up to 400 (25). Using the Excel randomization function a random sample of 20 Slovenian pharmacies was selected from a list of all Slovenian pharmacies. Thus 20 patients per pharmacy were needed to be recruited. The selected pharmacies received a written notification about the study, its aims and design. In the notification, the interview time was scheduled in three months. Pharmacies were informed that all data would be anonymous and strictly confidential. They also had the right to refuse participation.

### Study procedure

Patient interviews were conducted by final-year pharmacy students (N = 10) who had undergone a one-day training course designed to familiarize them with the study. Interviewers approached patients leaving the pharmacy and filtered out anyone who did not pick up a prescription. Interviewers explained the study aims, assured anonymity, and requested consent. Each interview referred to one of the medicines that the patient received at the most recent pharmacy visit. If a patient received multiple prescription medicines, one was randomly selected. Some of the patients refused to participate, but we did not register their number.

The interviewers followed the interview guide, writing down patients’ answers to the open-ended questions verbatim. The patients were allowed to use any written material they obtained at the pharmacy (including the patient information leaflet) to answer the questions. The interviewer noted if the patient needed to consult the written material to answer correctly. At the end of the interview, the patients were thanked for their participation. All interviews took place in November and December 2013. After the completion of the interviews, the noted patients’ responses were checked for consistency with Summaries of Product Characteristics (SmPCs). If the patient response matched the SmPC (eg, medication purpose the patient stated was listed in the SmPC), the response was labeled as consistent.

### Scoring system

Three separate scores were calculated. The first score evaluated patients’ knowledge, based on their answers to the open-ended questions. Patients received points if they knew a specific counseling element and their response was consistent with the corresponding SmPC. The second score assessed the pharmacist counseling. Pharmacists received points if their patients stated they were explained or asked about a specific counseling element. The third score assessed the adequacy of information provided by physicians and pharmacists during treatment. Physicians and pharmacists received points if the patients thought they received adequate information about a specific counseling element. The same criteria were used for all three scores: each counseling element received 1 point, with a maximum of 10 points (Table 1).

**Table 1:** Scoring system for evaluation of patient knowledge about their prescriptions, pharmacist counseling at the most recent pharmacy visit, and adequacy of physician and pharmacist information during treatment. For each item, 1 was assigned if judged satisfactory, 0 otherwise, to yield a total maximum possible score of 10

Counseling element	Patient knowledge	Pharmacist counseling	Physician/pharmacist adequate informing
Medication purpose	1	1	1
Dose	1	1	1
Rate of application	1	1	1
Timing of medicine	1	1	1
Route of administration	1	1	1
Taking with meals	1	1	1
Duration of therapy	1	1	1
Recognition of medication effectiveness	1	1	1
Considerations	1	1	1
Adverse effects	1	1	1
Total	10	10	10

### Statistical analysis

Standard descriptive statistics measures (mean, standard deviation [SD], median [5th-95th percentile]) were used. Normality of distribution was tested by Smirnov-Kolmogorov test and appropriate nonparametric methods were applied. Differences in quantitative values between two groups were tested by a Mann-Whitney test with Bonferroni correction. Differences in categorical variables were tested using χ^2^ test.

A multiple linear regression was performed to determine factors associated with patients’ knowledge. Therefore, the patients’ knowledge score was set as a dependent variable. The patient’s prescription type, number of current medicines, sex, age, education, income, assessment of current health status, assessment of pharmacist counseling, pharmacist counseling score, and physician/pharmacist informing score were used as factors. Dummy variables were used in cases of categorical variables with more than two categories. First, bivariate correlations between the factors and patient knowledge score were calculated. Those with non-significant correlations were excluded from further analysis. The remaining factors were entered into the regression model. The forced entry method of regression was used (SPSS: Enter method). Multicollinearity was examined by the variance inflation factors. Statistical analysis was performed in Microsoft Excel 2010 and SPSS v. 22 (26,27). A significance level below 0.05 was considered statistically significant.

## Results

All twenty contacted pharmacies agreed to participate in the study. Twelve pharmacies were from an urban and eight pharmacies from a rural setting.

### Sociodemographic data

On the 1-5 rating scale (1 = poor, 5 = excellent), patients assessed their current health status with the median [5th-95th percentile] of 3 [1.8-5]. The median of the current number of prescription medicines was 3 [1-8]. According to the Anatomical Therapeutic Chemical classification, the medicines chosen for the interviews were predominately from group C (cardiovascular, N = 94, 24%), N (nervous system, N = 65, 16%), and A (alimentary tract and metabolism, N = 51, 13%). There was a roughly equal number of new and regular prescriptions (149 vs 147) and 103 refill prescriptions. One patient did not know the prescription type (Table 2).

**Table 2:** Patients’ sociodemographic data

Patients’ characteristic	
**Sex, n (%)**	
female	215 (54)
male	183 (46)
missing data	2 (1)
**Education, n (%)**	
primary school or less	71 (18)
secondary school	228 (57)
college	53 (13)
university or more	41 (10)
missing data	7 (2)
**Monthly income in** EUR, **n (%)**	
less than 650	175 (44)
650-800	64 (16)
800-1000	49 (12)
1000-1300	33 (8)
more than 1300	22 (6)
missing data	57 (14)
**Age, median (range), n (%)**	
age in years	57 (12-92)
missing data	8 (2)

### Patients’ knowledge

**Figure 1:**
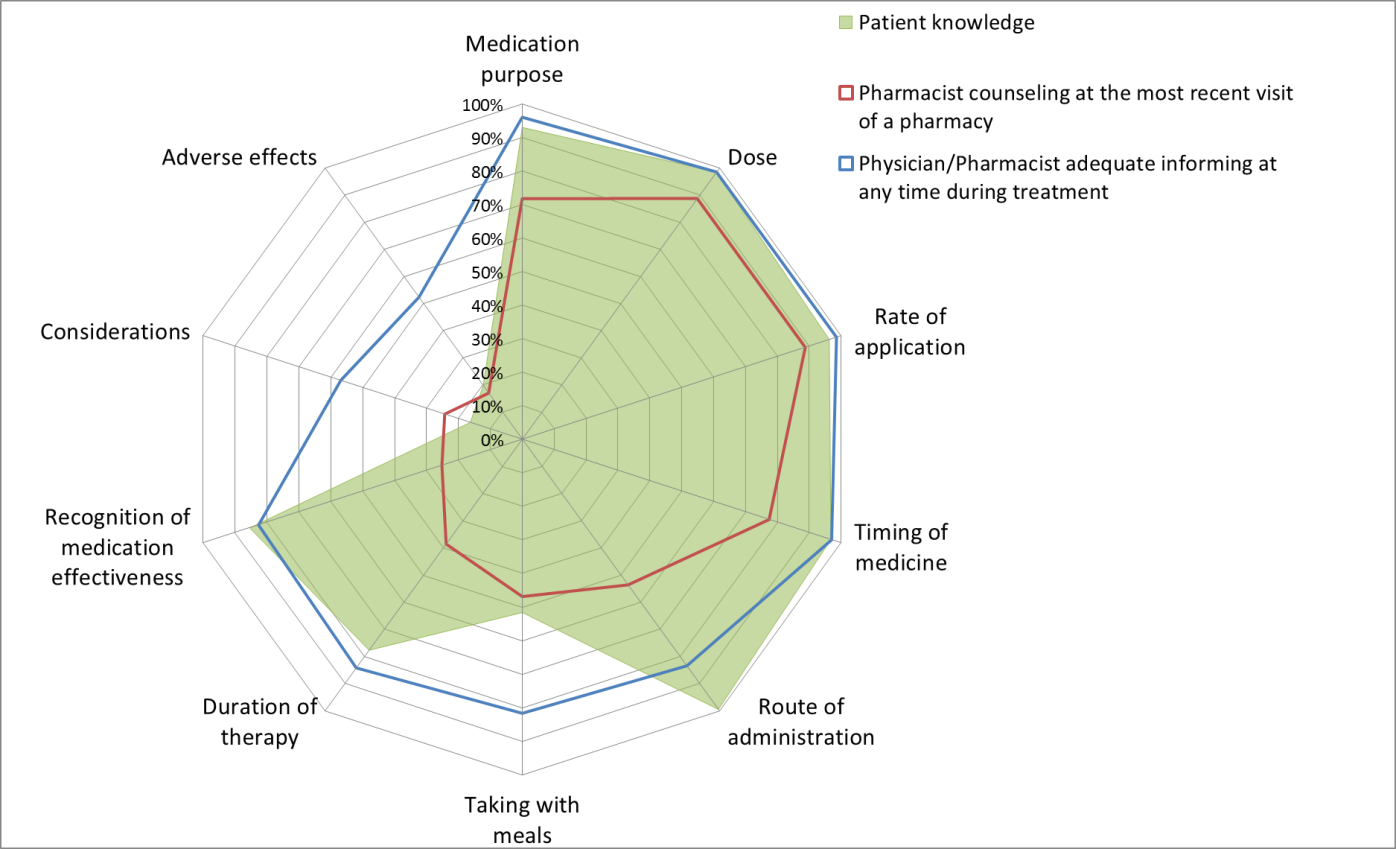
Patient knowledge of different counseling elements, pharmacist counseling at patients’ most recent visit to the pharmacy, and physician/pharmacist adequate informing. For each counseling element, the percentage of patients who had knowledge of a counseling element, the percentage of patients who stated they were offered information or verification for a counseling element at their most recent visit to the pharmacy, and the percentage of patients who thought that they were adequately informed about a counseling element during their treatment are shown.

Most patients were familiar with their medication purpose (N = 370, 93% of valid responses), dose (N = 390, 99%), application rate (N = 384, 96%), timing (N = 386, 97%), and administration route (N = 397, 100%). Fewer, but still a high percentage of patients knew how to determine the therapy duration (N = 310, 78%) and to recognize medication effectiveness (N = 341, 86%). 358 patients answered the question about taking their medicine with meals; however, 150 gave responses that were not consistent with the corresponding SmPCs. The most common mistake (92 out of 150 patients) was the answer that the medicine should be taken before or after a meal or even on an empty stomach when the SmPC stated that medicine could be taken irrespective of food intake. While this was not a pharmacotherapy concern, it represented a burden for patients who were unnecessarily preoccupied with taking their medicine with meals. Sixty-five patients (16%) knew about considerations. Mostly, they were familiar with contraindications (N = 16), interactions with alcohol (N = 11), and interactions with food (N = 10). Only 79 patients (20%) knew something about the adverse effects of their medicine. Most knew the possible adverse effects (N = 73), and a few knew how to prevent or minimize adverse effects (N = 10) (Figure 1). Nineteen patients (5%) used the written material they obtained at the pharmacy to answer the questions. Most used it for the question about adverse effects. The average score ± standard deviation for patient knowledge was 7.3 ± 1.2 points.

Table 3 shows the factors that significantly correlated with patients’ knowledge and were thus used in the regression model. The model explained 14.0% of variance of patients’ knowledge (R = 0.375, N = 378, *P* < 0.001). All variance inflation factors were below 1.2, indicating minor multicollinearity among the factors. The strongest factor associated with patient knowledge was provision of adequate informing from the physicians or pharmacists (β = 0.259, 6.0% of variance explained). Other statistically significant factors were age (β = -0.149, 1.5% of variance explained), prescription type (β = -0.104, 1.0% of variance explained), and education (β = 0.100, 0.9% of variance explained). Patients with refill prescriptions displayed lower level of knowledge than other patients. Patients with university or higher education demonstrated better knowledge scores. Other factors were not significantly associated with patients’ knowledge.

**Table 3:** Multivariate regression of patient knowledge score on patients’ sociodemographic data, pharmacist counseling score, and physician/pharmacist informing score. Factors that showed significant bivariate correlation with patient knowledge were entered into the regression model

Factor	B	95% confidence interval	Standardized β	*P*
(Constant)	6.468	5.804, 7.132		
Prescription type	-0.257	-0.503, -0.010	-0.104	0.042
Number of current medicines	-0.019	-0.064, 0.025	-0.046	0.395
Sex	0.139	-0.071, 0.349	0.064	0.195
Age	-0.009	-0.016, -0.002	-0.149	0.011
Education	0.352	0.005, 0.699	0.100	0.047
Pharmacist counseling	-0.007	-0.047, 0.034	-0.017	0.744
Physician/pharmacist adequate informing	0.154	0.094, 0.214	0.259	<0.001

### Pharmacist counseling at the most recent visit to the pharmacy

Patients reported whether the pharmacist explained or checked the patients’ knowledge of a specific counseling element at their most recent visit to the pharmacy (Figure 1). Pharmacists most often discussed medication purpose (N = 287, 72% of valid responses), dose (N = 354, 89%), application rate (N = 354, 89%), and timing (N = 308, 77%). About half of the patients stated they received information or verification about administration route (N = 214, 54%) and dosage time in relation to food (N = 187, 47%). Other information was rarely offered. Ninety-seven (24%) patients were given information on at least one consideration. Similarly, 68 (17%) patients were counseled on adverse effects. 31 patients (8%) claimed they received no counseling on any of the counseling elements. Patients assessed pharmacist counseling on a 5-point rating scale (1 = poor, 5 = excellent). The average rating was 4.5 ± 0.8.

With respect to prescription type, most counseling was performed for patients with new prescriptions. The difference in scores for pharmacist counseling (new vs regular, new vs refill) were significant (*P* = 0.03 and *P* = 0.003, respectively). There were no significant differences in pharmacist counseling scores between regular and refill prescriptions (*P* = 0.53). Overall, the average score for pharmacist counseling was 5.3 ± 2.7 points.

**Figure 2:**
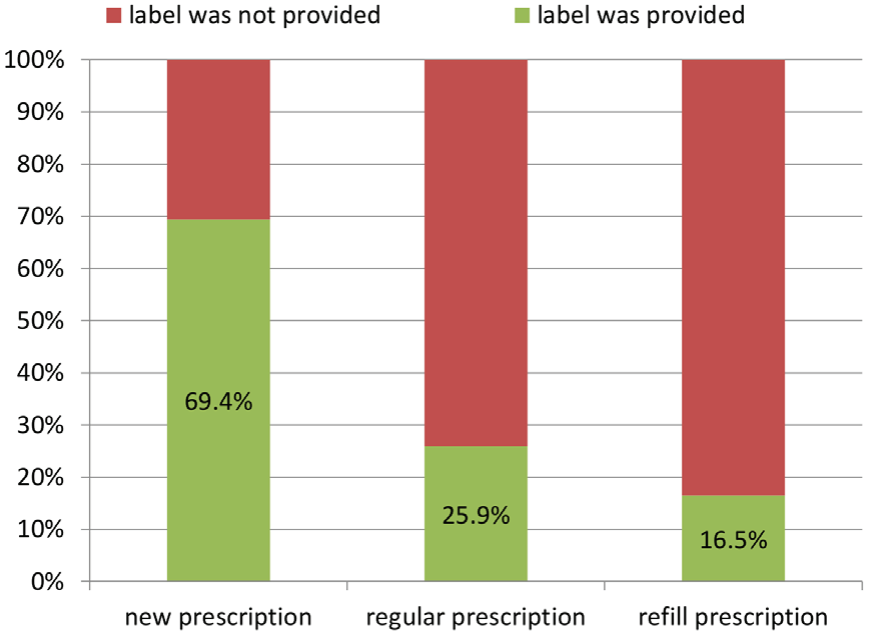
Pharmacists’ provision of medication labels according to the prescription type. Numbers represent the percentages of patients who were provided medication labels at their most recent pharmacy visit.

Pharmacists provided medication labels for 157 patients (39%). 6 of them could not read the pharmacist’s handwriting. Patients often commented that they did not need a medication label, because they already were familiar with their medicines. A small proportion of patients who had previously used their medicines were given medication labels (38 out of 147 with regular prescriptions, 17 out of 103 with refill prescriptions). On the other hand, 69% (102 out of 147) of patients with a new prescription received a medication label (Figure 2). The differences in label provision were significant (χ^2^ = 89.2, *P* < 0.001).

### Adequate informing by physician and pharmacist at any time during treatment

Almost all patients were satisfied with information on medication purpose, dose, application rate, and timing. Most felt that they were adequately informed about the administration route, taking of the medicine with meals, therapy duration, and the way to recognize medication effectiveness. However, about half of the patients wanted more information about adverse effects and medicine considerations. The average score for physician and pharmacist informing was 8.2 ± 2.4 points (Figure 1).

## Discussion

The results of this study suggest that Slovenian patients understand basic information about their prescription medicines; however, they lack knowledge about medication safety. This lack of knowledge can be attributed to pharmacist counseling, which, according to patients’ assessment, mainly focuses on medication administration and neglects medication considerations or adverse effects.

### Patients’ knowledge

Patients showed consistent knowledge about medication purpose, dose, application rate, timing, and administration route. Thus, a large proportion of patients knew how to administer their medicine. Other studies that assessed patient knowledge of prescription medicines also showed the highest patients’ scores in these areas (18,20,21).

In contrast to high levels of knowledge about administration of medicines, patients in this study showed a serious knowledge deficiency about medicine considerations and adverse effects. Patients were considered knowledgeable about a counseling element if they could name at least one item (eg, one adverse effect). Despite this lenient criterion, only one out of six patients knew something about considerations and one out of five patients knew something about adverse effects. Other studies that evaluated patients’ level of knowledge about their prescription found similar results (7,20,28). This study showed that lack of knowledge could be attributed to the deficiency of pharmacist counseling.

The results of the multiple regression analysis indicated that age was negatively associated with patient knowledge. This could be attributed to differences in education levels, diminishing cognitive capabilities, differences in values, psychosocial factors, and possible communication impairments (12,18). Thus, pharmacists should tailor their counseling to elderly patients (12,29). Patients with university or higher education showed better scores for patient knowledge, which is consistent with findings from similar studies (7,8,20,21). It has been shown that patients with higher education better understand counseling about their prescription medication (20).

### Medication counseling

The level of patients’ knowledge about their prescription medicines can be better understood by examining the counseling they received. Patients indicated they were frequently given directions for medication use, whereas information on considerations and adverse effects was seldom provided. Several studies report omission of drug safety issues during counseling (2,22,23,30). This negligence results in knowledge deficiency and a demand for more information. In this study, 43% of patients felt that they were inadequately informed about considerations and 48% felt the same about adverse effects. Similarly, Nair et al (31) found that patients in focus groups expressed frustration about not getting enough information about adverse effects and risks.

Properly informed patients are likely to feel more control over and less apprehension about their medication use. They are more attentive to adverse effects, which they detect more quickly than patients who do not receive adequate information (29). In this respect, early contact with health care providers can prevent serious adverse effects. Additionally, patients who are not told about possible adverse effects are more likely to be intimidated when reading them in the patient information leaflet (29). Studies by Lamb et al (32) and Howland et al (33) also indicate that counseling patients about adverse effects does not increase the incidence of adverse effects or decrease therapy adherence. Hence, pharmacists should be advised to pay greater attention to counseling about drug safety without creating an information overload (29).

This study indicated that patients knew more about their prescription medicines than what they learned at their most recent pharmacy visit. The largest discrepancies between patient knowledge and perceived pharmacist counseling were observed in cases of recognizing medication effectiveness, route of administration, and taking of medicines with meals. For example, 86% of the patients knew how to recognize their medication effectiveness, while only 25% indicated they received counseling about this topic at their most recent pharmacy visit. In this respect, adequate informing by the physician or pharmacist during treatment seemed to have a greater effect on patient knowledge than did pharmacist counseling at the most recent pharmacy visit. The results of the regression analysis, in which physician and pharmacist informing was a significant factor associated with patient knowledge, support this finding.

Consistent with other studies, patients who collected new prescriptions received significantly more counseling than those who collected regular or refill prescriptions (2,30). This finding might be explained by lack of patients’ interest and their belief that medication counseling for regular or refill prescriptions was unnecessary (2,22). Another reason might be that the pharmacist assumed that patients with chronic conditions already knew how to properly use their medicines (22). This study did not support this assumption, as the prescription type was a significant factor associated with medication knowledge. The average patient knowledge scores for new, regular, and refill prescriptions were 7.5, 7.4, and 7.1, respectively. Patients with refill prescriptions thus demonstrated significantly lower knowledge scores. Since these patients usually see their physician only once a year, it is the responsibility of a community pharmacist to follow these patients and provide adequate counseling. This study suggests community pharmacists should improve their counseling for patients with refill prescriptions, thus enhancing their knowledge.

Many patients in this study stated they did not receive counseling about important medication information at their most recent pharmacy visit. Nevertheless, patients rated pharmacist counseling 4.5 out of 5 on average, and 63% of those surveyed assessed counseling with the highest possible score. Despite expressing a need for further information, especially about safety issues, patients seemed satisfied with the pharmacist counseling they received. Patient satisfaction studies reported similar results (34-36). These findings might be explained by patients’ tendency to accept their care until something unacceptable happens (35). Patients are also generally reluctant to express criticism or concern (37).

Medication labels were provided for 39% of patients, with significant differences relating to prescription type. Low rates of medication label provision for the regular and refill prescriptions are understandable. Medication labels in Slovenia mostly contain information on dose and dosing interval, and patients are usually familiar with this information. However, 31% of patients with new prescriptions did not receive medication labels. Providing written information in addition to verbal counseling enhances patient knowledge and encourages safer medication use (29). Furthermore, the “Rules on the Classification, Prescribing and Dispensing of Medicinal Products for Human Use,” which are valid in Slovenia, declare that each dispensed medicine should be supplied with a label (38). In this respect, pharmacists can improve their provision of medication labels and thus fulfill their professional and legal obligations.

### Limitations and conclusion

The researchers checked the consistency of patient responses with SmPCs. To verify the accuracy of patients’ recollections, more information on patients and their diseases would be needed. Furthermore, patients may underestimate the types of information they get since they have forgotten the actual information they received (22). Nevertheless, Schommer et al (24) found that patient surveys were a useful way to collect data about counseling, though they stipulated that questions must be carefully worded. In summary, patients knew basic information about the administration of their prescription medicines, but they lacked knowledge about medication safety. This deficiency could be attributed to counseling, which primarily focused on medicine use instructions.
