# Supplementary material for: Contribution of Slovenian community pharmacist counseling to patients’ knowledge about their prescription medicines: a cross-sectional study
Source: Croat Med J. 2015 Feb;56(1):41–9. doi: 10.3325/cmj.2015.56.41 (PMC4364351; doi:10.3325/cmj.2015.56.41)
Supplement: Supplementary material [file CroatMedJ_56_s002.pdf]

Questionnaire

University of Ljubljana – Faculty of Pharmacy

Medication data

Medication name	
Dosage strength	
Prescription type	□ new<br>□ regular<br>□ refill<br>□ info:
How many different prescription medicines are you currently taking?<br>*[different propriety names, including those received today]*	

Prescription medicine information

1. **What do you take this medication for?**

[specify patient's verbatim response]

□ does not know □ read the information □ other:

Did the pharmacist **inform** you about the purpose of this medication **at your last visit** of this pharmacy?	□ yes □ no
[if NO]: Although the pharmacist hasn't informed you about the purpose of this medication *[at your last visit of this pharmacy]*, did he or she **specifically ask** you whether you already know this information?	□ yes □ no
Do you think that, **during your treatment**, the **physicians and pharmacists** you encountered **adequately informed** you about the purpose of this medication?	□ yes □ no

2. **How often should you take this medication and how much should you take at that time?** *[dose, application rate]*

______x a	□ day<br>□ week<br>□ month<br>□ other: __________	_____	□ tablets<br>□ capsules<br>□ spoons<br>□ suppositories	□ ovules<br>□ drops<br>□ inhalations<br>□ other: ____________

□ does not know □ read the information □ other:

Did the pharmacist **inform** you how often should you take this medication and how much should you take **at your last visit** of this pharmacy?	□ yes □ no
[if NO]: Although the pharmacist hasn't informed you how often should you take this medication and how much should you take *[at your last visit of this pharmacy]*, did he or she **specifically ask** you whether you already know this information?	□ yes □ no

Do you think that, **during your treatment**, the **physicians and pharmacists** you encountered **adequately informed** you about the how often should you take this medication and how much should you take?	□ yes □ no

1. **When should you take this medication?** *[application timing]*

□ morning<br>□ noon<br>□ evening	□ other:
□ does not know □ read the information □ other:	

Did the pharmacist **inform** you when should you take this medication **at your last visit** of this pharmacy?	□ yes □ no
[if NO]: Although the pharmacist hasn't informed you when should you take this medication *[at your last visit of this pharmacy]*, did he or she **specifically ask** you whether you already know this information?	□ yes □ no
Do you think that, **during your treatment**, the **physicians and pharmacists** you encountered **adequately informed** you when should you take this medication?	□ yes □ no

2. **How should you administer this medication?** *[route of administration]*

□ swallow<br>□ dissolve in mouth<br>□ under the tongue<br>□ apply onto the skin<br>□ stick onto the skin	□ ear<br>□ nose<br>□ eye<br>□ anus<br>□ vagina	□ inhale<br>□ other:
□ does not know □ read the information □ other:		

Did the pharmacist **inform** you how should you administer this medication **at your last visit** of this pharmacy?	□ yes □ no
[if NO]: Although the pharmacist hasn't informed you how should you administer this medication *[at your last visit of this pharmacy]*, did he or she **specifically ask** you whether you already know this information?	□ yes □ no
Do you think that, **during your treatment**, the **physicians and pharmacists** you encountered **adequately informed** you how should you administer this medication?	□ yes □ no

3. **How should you take this medication with meals?** *[taking with meals]*

□ before a meal<br>□ during a meal<br>□ after a meal	□ fasting<br>□ irrespective of meal<br>□ other:
□ does not know □ read the information □ other:	

Did the pharmacist **inform** you how should you take this medication with meals **at your last visit** of this pharmacy?	□ yes □ no
[if NO]: Although the pharmacist hasn't informed you how should you take this medication with meals *[at your last visit of this pharmacy]*, did he or she **specifically ask** you whether you already know this information?	□ yes □ no
Do you think that, **during your treatment**, the **physicians and pharmacists** you encountered **adequately informed** you how should you take this medication with meals?	□ yes □ no

4. **How long should you take this medication?** *[duration of therapy]*

[specify patient's verbatim response]		
□ does not know	□ read the information	□ other:

Did the pharmacist **inform** you how long should you take this medication **at your last visit** of this pharmacy?	□ yes	□ no
[if NO]: Although the pharmacist hasn't informed you how long should you take this medication *[at your last visit of this pharmacy]*, did he or she **specifically ask** you whether you already know this information?	□ yes	□ no
Do you think that, **during your treatment**, the **physicians and pharmacists** you encountered **adequately informed** you how long should you take this medication?	□ yes	□ no

5. **How will you know that the medication is working?** *[recognition of medication effectiveness]*

[specify patient's verbatim response]		
□ does not know	□ read the information	□ other:

Did the pharmacist **inform** you how will you know that the medication is working **at your last visit** of this pharmacy?	□ yes	□ no
[if NO]: Although the pharmacist hasn't informed you how will you know that the medication is working *[at your last visit of this pharmacy]*, did he or she **specifically ask** you whether you already know this information?	□ yes	□ no
Do you think that, **during your treatment**, the **physicians and pharmacists** you encountered **adequately informed** you how will you know that the medication is working?	□ yes	□ no

6. **What should you pay attention to while taking this medication?**

[specify patient's verbatim response]		
□ does not know	□ read the information	□ other:

Did the pharmacist **inform** you what should you pay attention to while taking this medication **at your last visit** of this pharmacy?	□ yes	□ no
[if NO]: Although the pharmacist hasn't informed you what should you pay attention to while taking this medication *[at your last visit of this pharmacy]*, did he or she **specifically ask** you whether you already know this information?	□ yes	□ no

Do you think that, **during your treatment**, the **physicians and pharmacists** you encountered **adequately informed** you what should you pay attention to while taking this medication? □ yes □ no

7. **What do you know about adverse effects of this medication?**

[specify patient's verbatim response]

□ does not know □ read the information □ other:

Did the pharmacist **inform** you about adverse effects of this medication **at your last visit** of this pharmacy? □ yes □ no

[if NO]: Although the pharmacist hasn't informed you about adverse effects of this medication *[at your last visit of this pharmacy]*, did he or she **specifically ask** you whether you already know this information? □ yes □ no

Do you think that, **during your treatment**, the **physicians and pharmacists** you encountered **adequately informed** you about adverse effects of this medication? □ yes □ no

8. **Did the pharmacist tell you any other important information about this medication at your last visit of this pharmacy?** □ yes □ no
   [if YES]: What else did he tell you?

[specify patient's verbatim response]

9. **Did the pharmacist provide medication label at your last visit of this pharmacy?** □ yes □ no
   [if YES]: Could you please explain the medication label?

□ cannot read the label
□ reads:

10. **Did the pharmacist provide any other written information at your last visit of this pharmacy?** □ yes □ no
    [if YES]: Which?

[specify patient's verbatim response]

Sociodemographic Data

Sex	□ male<br>□ female
Age	__________<br>□ refused to answer
Education	□ primary school/unfinished primary school<br>□ secondary school<br>□ college<br>□ university or higher<br>□ refused to answer
Assessment of pharmacist counselling at your last visit	poor [1] fair [2] good [3] very good [4] excellent [5]<br>□ refused to answer
Income	□ less than 650 €/without personal income<br>□ 650 – 800 €<br>□ 800 – 1000 €<br>□ 1000 – 1300 €<br>□ more than 1300 €<br>□ refused to answer
Assessment of current health status	poor [1] fair [2] good [3] very good [4] excellent [5]<br>□ refused to answer
